# Urine Cell-Free DNA Integrity as a Marker for Early Prostate Cancer Diagnosis: A Pilot Study

**DOI:** 10.1155/2013/270457

**Published:** 2013-02-13

**Authors:** Valentina Casadio, Daniele Calistri, Samanta Salvi, Roberta Gunelli, Elisa Carretta, Dino Amadori, Rosella Silvestrini, Wainer Zoli

**Affiliations:** ^1^IRCCS Istituto Scientifico Romagnolo per lo Studio e la Cura dei Tumori (IRCCS IRST), Via P. Maroncelli 40,47014 Meldola, Italy; ^2^Department of Urology, Morgagni Pierantoni Hospital, Via C. Forlanini 34, 47121 Forli, Italy

## Abstract

Circulating cell-free DNA has been recognized as an accurate marker for the diagnosis of prostate cancer, whereas the role of urine cell-free DNA (UCF DNA) has never been evaluated in this setting. It is known that normal apoptotic cells produce highly fragmented DNA while cancer cells release longer DNA. We thus verified the potential role of UCF DNA integrity for early prostate cancer diagnosis. UCF DNA was isolated from 29 prostate cancer patients and 25 healthy volunteers. Sequences longer than 250 bp (*c-Myc*, *BCAS1*, and *HER2*) were quantified by real-time PCR to verify UCF DNA integrity. Receiver operating characteristic (ROC) curve analysis revealed an area under the curve of 0.7959 (95% CI 0.6729–0.9188). At the best cut-off value of 0.04 ng/**μ**L, UCF DNA integrity analysis showed a sensitivity of 0.79 (95% CI 0.62–0.90) and a specificity of 0.84 (95% CI 0.65–0.94). These preliminary findings indicate that UCF DNA integrity could be a promising noninvasive marker for the early diagnosis of prostate cancer and pave the way for further research into this area.

## 1. Introduction

Early diagnosis plays an important role in the treatability of patients with different tumor types in terms of disease-free and overall survival. Prostate cancer has a high incidence and represents the second cause of death from cancer in men after lung cancer. Early diagnosis is thus essential, especially in view of the slow natural history of the disease and its potential curability in the initial hormone-dependent phase. Non-invasive diagnostic procedures have a higher patient compliance and a lower cost than invasive screening programs. At present, the only noninvasive approach currently used for the diagnosis of prostate cancer is the determination of PSA (prostate-specific antigen) in blood, which has been shown to reduce prostate cancer mortality. However, the use of PSA has recently been questioned because of its low accuracy, especially in terms of specificity. False positive results lead to overtreatment in individuals, with consequently higher healthcare costs and psychological distress [1–5]. Although great efforts have been made to improve the diagnostic accuracy of PSA, the search continues for new molecular markers, proteins, or genetic and epigenetic alterations [6, 7] to be used in this setting. 

 New accurate and cost-effective diagnostic approaches are needed to enhance or replace standard techniques for prostate cancer diagnosis. Cell-free nucleic acids have proven useful for early cancer diagnosis and positive results have also been published on serum and plasma cell-free DNA and RNA as sources of tumor-specific markers [8, 9]. Circulating cell-free DNA has been shown to play an important diagnostic role in colon [10] and lung cancer [11], and a number of studies have also highlighted its potential usefulness in prostate cancer [12–14]. Urine cell-free (UCF) DNA as a source of tumor biomarkers has not been adequately investigated in prostate cancer and only a few recent studies have discussed its potential importance for early bladder cancer diagnosis [15–17].

 It has been shown that DNA from normal apoptotic cells is highly fragmented, whereas DNA from necrotic cancer cells maintains its integrity [18]. Taking this into account and also considering recent results on bladder cancer highlighting the importance of UCF DNA integrity for early diagnosis [15], we investigated the ability of this marker to distinguish between prostate cancer patients and healthy individuals by analyzing UCF-DNA fragments longer than 250 bp in 3 regions is known to be frequently amplified in solid tumors, including prostate cancer: *c-Myc* (8q24.21), *HER2* (17q12.1), and *BCAS1* (20q13.2) [19–21].

## 2. Materials and Methods

### 2.1. Case Series

This pilot study was composed of 54 individuals, 29 at first diagnosis of prostate cancer and 25 healthy individuals (control group) matched to patients for age. Subjects with previous or concomitant urogenital diseases or cancers were excluded from the study. Healthy individuals underwent transrectal ultrasound (TRUS) to exclude the presence of prostate cancer. Participants were recruited from the Department of Urology of Morgagni, Pierantoni Hospital (Forli) and all provided written informed consent to take part in the study, which was reviewed and approved by the local Ethics Committee. Median age was 65 years for patients and 66 for healthy individuals. All patients underwent radical prostatectomy. The Gleason score and pathological stage were evaluated after surgical removal of the tumor. Twelve patients had a Gleason score of ≤6 and 17 patients had a score of >6. Two patients had pT2a tumors, 14 pT2b, 10 pT3a, and 3 pT3c. The median PSA value was 7.5 (range 3.19–33) (Table 1). 

### 2.2. Urine Collection

First-morning-void urine samples were collected for UCF DNA analysis. For prostate cancer patients, specimens were collected before radical prostatectomy. All patients and controls were instructed to give clean-catch urine samples, which were maintained at 4°C for a maximum of 3 hours. Thirty milliliter aliquots of urine were centrifuged at 850 g for 10 minutes and the supernatants were transferred to cryovials and immediately stored at −80°C until use.

### 2.3. UCF DNA Analysis

DNA was extracted and purified from 2 mL of supernatant by Qiamp DNA minikit (Qiagen, Milan, Italy) according to the manufacturer's instructions. At the same time, DNA was extracted from a human bladder cancer cell line (MCR) using the same minikit and quantified by spectrophotometry (NanoDrop ND-1000, Celbio, Milan, Italy).

 Real-time PCR reactions were carried out by Rotor Gene 6000 detection system (Corbett Research, St. Neots, UK) using IQ SYBR green (Biorad, Milan, Italy). Sequences longer than 250 bp corresponding to 3 oncogenes were analyzed as follows: *c-Myc* (locus 8q24.21, amplification product 264 bp), *BCAS1* (locus 20q.13.2, amplification product 266 bp), and *HER2* (locus 17q12.1, amplification product 295 bp). A short 125 bp fragment of *STOX1* (locus 10q21.3) was analyzed to check for potential PCR inhibition. Primer sequences were as follows: *c-Myc* fw TGGAGTAGGGACCGCATATC, rev ACCCAACACCACGTCCTAAC; *BCAS1* fw GGGTCAGAGCTTCCTGTGAG, rev CGTTGTCCTGAAACAGAGCA; *HER2* fw CCAGGGTGTTCCTCAGTTGT, rev TCAGTATGGCCTCACCCTTC; *STOX1* fw GAAAACAGGGCAGCAAGAAG, rev CAGACAGCATGGAGGTGAGA. PCR conditions for the oncogenes were as follows: 95°C for 3 minutes followed by 45 cycles at 94°C for 40 seconds, 56°C for 40 seconds, and 72°C for 1 minute. PCR conditions for the short *STOX1* sequence were as follows: 95°C for 90 seconds followed by 45 cycles at 94°C for 40 seconds and 54°C for 1 minute. All real-time PCR reactions were performed in duplicate on 10 ng of each UCF DNA sample. Various amounts of DNA from the MCR cell line (0.01, 0.1, 1, 5, 10, and 20 ng) were also analyzed to construct a standard curve. The UCF DNA value for each sample was obtained by Rotor Gene 6000 detection system software using standard curve interpolation. The analysis was repeated if the difference between duplicate samples was greater than 1 cycle threshold. The final UCF DNA integrity value was obtained by summing the three oncogene values. Real-time experiments were performed independently in duplicate on the same 8 samples to test assay variability. The coefficients of variation (CV) were then calculated for *c-Myc*, *HER2*, *BCAS*, and *STOX1*. Real-time PCR analyses were performed in accordance with MIQE guidelines (remarks to the MIQE checklist are included as Supplementary Table 1 available online at http://dx.doi.org/10.1155/2013/270457) [22].

### 2.4. Statistical Analysis

The relationship between UCF DNA integrity values in the two subgroups was analyzed using a nonparametric ranking statistic test. The most discriminating cut-off values between healthy individuals and cancer patients were identified using receiver operating characteristic (ROC) curve analysis. True positive rates (sensitivity) were plotted against false positive rates (1-specificity) for all classification points. Accuracy was measured by the area under the ROC curve (AUC), which represents an average probability of correctly classifying a case chosen at random. Study endpoints were sensitivity (the proportion of cancer patients who were correctly identified by the test or procedures) and specificity (the proportion of healthy individuals who were correctly identified), with their 95% confidence intervals (CIs) [23]. *P* values <0.05 were considered statistically significant. Statistical analyses were performed using SPSS statistical software (version 12.0, SPSS GmbH Software).

## 3. Results

Total free DNA showed a median value of 6 ng/*μ*L (range 2–36 ng/*μ*L). There was no statistically significant difference between total urine cell free DNA in cancer patients and healthy individuals (*P* = 0.1200 Wilcoxon-Mann-Whitney test).

The ROC curve for total free DNA showed an AUC of 0.6262 (Supplementary Figure 1). UCF DNA integrity analysis was feasible and results were evaluable for all 54 individuals. The 125 bp *STOX1* sequence was amplified in all samples, thus excluding the presence of PCR inhibitors. Values showed a wide variability in both healthy individuals and cancer patients, with a partial overlapping. However, median values were significantly lower (about 20-fold, *P* = 0.0004) in healthy than in cancer patients (Table 2). 

**Figure 1:**
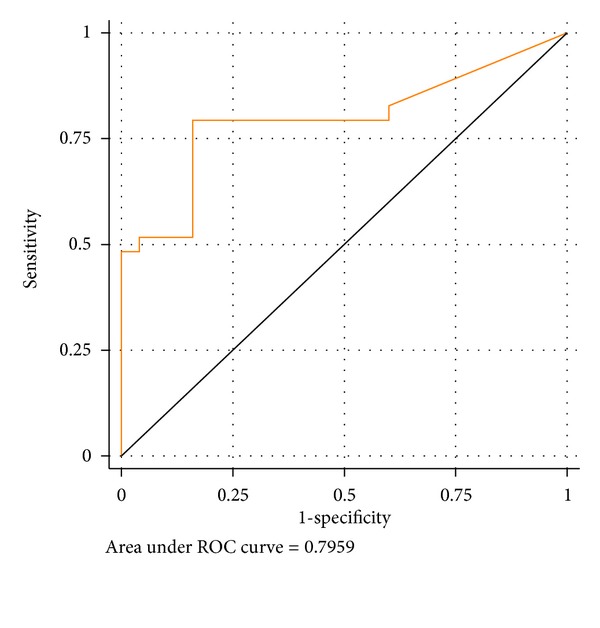
ROC curve of UCF DNA integrity.

ROC curve analysis of UCF DNA integrity showed an AUC of 0.7959 (0.6729–0.9188) for healthy individuals and cancer patients (Figure 1). Detailed analysis of sensitivity and specificity highlighted a different accuracy for the various UCF DNA cut-off values, with a sensitivity of 0.79 for 0.03 and 0.04 cutoffs which decreased at the highest cut-off values and a specificity of 0.84 which remained consistent for all cutoffs from 0.03 to 0.06 (Table 3). 

UCF DNA integrity did not significantly vary between younger (<70 years) and older individuals (data not shown). The analysis of UCF DNA as a function of tumor characteristics did not highlight any significant differences between patients with a Gleason score of ≤6 and those with a score of >6 or between pT2 and pT3 patients (data not shown).

 The median PSA value in the patients analyzed was 7.5. Sixteen patients had a PSA value between 4 and 10, considered as a “gray zone,” and 12 of these had a positive UCF DNA result, with a sensitivity of 0.75 (data not shown). We also performed ROC curve analysis for each gene in order to verify the role of single genes in determining test accuracy; AUC values were as follows: 0.7862 for *c-MYC* (95% CI: 0.6595–0.9129) 0.7779 for *HER2* (95% CI: 0.6625–0.8934) and 0.7076 for BCAS (95% CI: 0.5771–0.8381) (Table 4). However, the AUC values observed for the different genes were not statistically different (chi-square test).

## 4. Discussion

In recent years increasing efforts have been made to identify new diagnostic markers and to develop noninvasive diagnostic approaches that can be used additionally or as an alternative to common invasive tests to increase diagnostic accuracy for solid tumors. Important results have been obtained for lung [11] and colon cancer [10]. In a urological setting, studies performed to improve the early noninvasive diagnosis of prostate cancer have highlighted the usefulness of specific DNA alterations (methylation or mutations) in blood to identify cancer patients [12, 24, 25], but the potential of cell-free DNA in urine has been never investigated.

 Starting from recent results on the diagnostic relevance of urine cell free DNA for bladder cancer [15–17], we extended the research to prostate cancer, hypothesizing that long DNA in urine may have two different origins: necrotic bladder cancer cells [15] or necrotic prostate cancer cells. We excluded that DNA fragments passing through the glomerular filtration barrier could influence our analysis because these fragments are short, as demonstrated by SU and coworkers [26]. Our results also showed that urine DNA integrity is capable of distinguishing between prostate cancer patients and healthy individuals with an accuracy of about 80%, similar to that observed for bladder cancer. Such findings are seemingly in contrast to those of Ellinger and coworkers who found a positive correlation between prostate cancer and the presence of short DNA fragments in blood [25]. The difference between cell free DNA in urine or blood remains unclear and should be investigated by comparing DNA integrity determined in blood and urine samples from the same patients.

 We also analyzed the diagnostic accuracy of DNA integrity of three oncogenes (*c-MYC*, *HER2*, and *BCAS1*) which are known to be involved in the development of bladder cancer. A comparison of ROC curves revealed that *c-MYC* had the highest AUC, a finding supported by evidence that *c-MYC* is involved in prostate tumorigenesis [27]. Furthermore, literature data on CGH array and copy number alterations highlight a high frequency of gain at 8q24 region where the *c-MYC* oncogene maps [19, 28–30], which could explain the higher number of copies of long *c-MYC* fragments in urine samples from prostate cancer patients than in those from healthy individuals. Lower diagnostic accuracy was observed for *HER2* and decreased further for *BCAS1*, but the AUC values observed for the different genes were not significantly different.

 The main limitation of this potentially important diagnostic finding is that it was obtained from a pilot study on a relatively small number of individuals. However, our results are being validated in a large confirmatory study ongoing at our institute. The advantage of the proposed approach is that cell free DNA, as previously shown [15], can be easily detected in a very small amount of urine. Moreover, unlike protein or RNA, it has good stability and is an inexpensive noninvasive method whose results are obtainable in about two working days. In the future it could be used as a test on its own or, thanks to its high specificity, could help to unmask cases of false positive PSA, especially in the subgroup of individuals with *grey zone* PSA values, thus reducing the number of unnecessary invasive diagnostic tests (e.g., prostate biopsy) carried out. 

## 5. Conclusions

The results obtained in the present work indicate that urine cell-free DNA integrity is a potentially good marker for the early diagnosis of noninvasive prostate cancers, with an overall diagnostic accuracy of about 80%. This preliminary finding paves the way for confirmatory studies on larger case series.

## Supplementary Material

Supplementary Table: Remarks to MIQE checklist.
Supplementary Figure: Receiver operating characteristic (ROC) curve analysis of total urine cell free DNA quantified with Nanodrop ND-1000.

## Figures and Tables

**Table 1:** Case series.

	Number	Age (yrs)		Median PSA (range)	Gleason score		Pathological stage			
		<70	≥70		≤6	>6	pT2a	pT2b	pT3a	pT3c
Healthy individuals	25	15	10	—	—	—	—	—	—	—
Prostate cancer patients	29	20	9	7.5 ( 3.19–33)	12	17	2	14	10	3

**Table 2:** UCF DNA integrity in healthy individuals and prostate cancer patients.

	UCF DNA integrity (ng/*μ*L)			
	Number	Median values (range)	Mean values (range)	P*
Healthy individuals	25	0.007 (0–0.141)	0.023 (0–0.141)	0.0004
Prostate cancer patients	29	0.129 (0–5.379)	0.533 (0–5.379)	

*Wilcoxon-Mann-Whitney test.

**Table 3:** Sensitivity and specificity of different UCF DNA integrity cut-off values.

Cutoff (ng/*μ*L)	Sensitivity	Specificity
0.03		
*n *	23/29	21/25
Rate (95% CI)	0.79 (0.62–0.90)	0.84 (0.65–0.94)
0.04		
*n *	23/29	21/25
Rate (95% CI)	0.79 (0.62–0.90)	0.84 (0.65–0.94)
0.05		
*n *	19/29	21/25
Rate (95% CI)	0.66 (0.47–0.80)	0.84 (0.65–0.94)
0.06		
*n *	17/29	21/25
Rate (95% CI)	0.59 (0.41–0.74)	0.84 (0.65–0.94)

**Table 4:** Area under ROC curve for each single gene and for UCF DNA integrity.

	AUC (95% CI)	P*
*c-Myc*	0.7862 (0.6595–0.9129)	
*BCAS1*	0.7076 (0.5771–0.8381)	NS**
*HER2*	0.7779 (0.6625–0.8934)	
UCF DNA integrity	0.7959 (0.6729–0.9188)	

*Chi-square test.

**NS: not significant.
