# Supplementary material for: Urine Cell-Free DNA Integrity as a Marker for Early Prostate Cancer Diagnosis: A Pilot Study
Source: Biomed Res Int. 2013 Feb 13;2013:270457. doi: 10.1155/2013/270457 (PMC3586456; doi:10.1155/2013/270457)
Supplement: Supplementary file 2 [file 270457.f2.docx]

**Supplementary figure 1. ROC curve of total UCF DNA**
